# Non-canonical autophagy in aging and age-related diseases

**DOI:** 10.3389/fcell.2023.1137870

**Published:** 2023-02-23

**Authors:** Anita V. Kumar, Joslyn Mills

**Affiliations:** ^1^ Molecular Biology, Cell Biology and Biochemistry Department, Brown University, Providence, RI, United States; ^2^ Wheaton College, Biology Department, Norton, MA, United States

**Keywords:** non-canonical autophagy, aging, neurodegenerative diseasaes, secretory autophagy, LAP (LC3 associated phagocytosis), LANDO

## Abstract

Autophagy, one of the arms of proteostasis, influences aging and age-related diseases. Recently, the discovery of additional roles of autophagy-related proteins in non-canonical degradation and secretion has revealed alternative fates of autophagic cargo. Some of these non-canonical pathways have been linked to neurodegenerative diseases and improving the understanding of this link is crucial for their potential targetability in aging and age-related diseases. This review discusses recent investigations of the involvement of non-canonical autophagy players and pathways in age-related diseases that are now beginning to be discovered. Unraveling these pathways and their relation to classical autophagy could unearth a fascinating new layer of proteostasis regulation during normal aging and in longevity.

## 1 Introduction

Autophagy, the process of sequestration of damaged macromolecules and organelles, culminates with cargo being degraded in lysosomes. Based on the specificity of cargo selection and the mechanism of cargo delivery to the lysosome, the process has been sorted into various forms of autophagy (reviewed by Kaushik and Cuervo (2012); Abdrakhmanov et al. (2020)). Macroautophagy requires the conjugation of members of the ATG8 family, ubiquitin-like proteins including LC3s and GABARAPs, to phosphatidylethanolamine (PE) (Ichimura et al., 2000). This enables double-membrane vesicles termed autophagosomes to recruit ATG8 proteins, which mediate loading and maturation of cargo (Johansen and Lamark, 2020). More recently, autophagy-independent functions of ATG8 proteins have been discovered (reviewed by Galluzzi and Green (2019); Nieto-Torres et al. (2021a)). Some of these functions involve unconventional conjugation of ATG8 proteins to phosphatidylserine (PS) in addition to that of PE and incorporation of ATG8-PE/PS into single-membrane vesicles, a process known as Conjugation of ATG8 to Single Membranes (CASM) (Durgan et al., 2021). Additionally, post translational modifications such as phosphorylation of LC3B/ATG8 on Thr50 regulates directionality of autophagosome movement toward the cell periphery in mammalian cells and neurons (Nieto-Torres et al., 2021b) which could potentially influence the fate of autophagosomes. Several recent studies have highlighted these additional roles of ATG8 proteins leading to alternative fates of their cargo in degradation and secretion, together referred to as non-canonical autophagy (NCA) (reviewed by Codogno et al., 2011; Nieto-Torres et al., 2021a).

## 2 Forms of non-canonical autophagy

**FIGURE 1:**
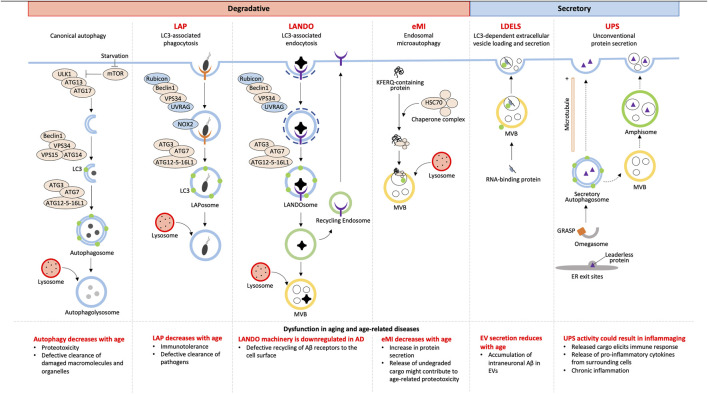
Overview of canonical and non-canonical autophagy and their dysfunctions in aging.

Although autophagy has always been accepted as a degradative process, not all cargoes from NCA culminate with lysosomal degradation. Owing to the alternative fates of cargoes, NCA can be either degradative or secretory (Figure 1).

### 2.1 Degradative autophagy

#### 2.1.1 LC3-associated phagocytosis

Combining the forces of phagocytosis with LC3 recruitment, LC3-associated phagocytosis (LAP) enhances the fusion of LC3-associated phagosomes (LAPosomes) with lysosomes to increase degradation and elimination of LAPosome-contained pathogens (reviewed Heckmann and Green, 2019; Herb et al., 2020). This is different than a branch of canonical selective autophagy, termed xenophagy, which targets cytosolic pathogens and other foreign material for degradation (reviewed by Sharma et al. (2018)). Although the fundamental LC3 conjugation machinery consisting of ATG7, ATG3, and a complex of ATG16L1, ATG5, and ATG12 are shared between xenophagy and LAP, the LAP pathway differs in the mechanism of induction, membrane PI(3)P generation, requirement for reactive oxygen species (ROS), conjugation of LC3 to single membranes, and regulation of associated genes (reviewed in Heckmann and Green (2019)).

Unlike canonical autophagy, LAP starts with phagocytosis that is initiated *via* receptors on the cell surface such as pattern recognition, IgG, and dead cell receptors (Sanjuan et al., 2007; Martinez et al., 2011). LAP and autophagy share the components of the PI3K complex, Beclin1, VPS15, and VPS34, for membrane PI(3)P generation, however, LAP PI3K complexes additionally require UVRAG and Rubicon which are necessary for downstream events such as LC3 recruitment (Martinez et al., 2015). Prior to LC3 conjugation, LAP requires NOX2-mediated ROS generation at the phagosome membrane which regulates phagosomal pH and signals that recruit the LC3 conjugation machinery ((Martinez et al., 2015); reviewed by Heckmann and Green (2019)). Phagosomes are then decorated with LC3 *via* CASM which requires the WD40 C-terminal domain of ATG16L1, which is a domain that is dispensable for ATG16L1’s role in canonical autophagy (Fletcher et al., 2018). Unlike in autophagy, LC3 lipidation occurs after cargo is selected and the phagosome is sealed (Sanjuan et al., 2007; Martinez et al., 2015), suggesting LC3’s role in cargo selection is unlikely, but rather LC3’s role in LAP is predominantly in phagosome-lysosome fusion (Martinez et al., 2015). Subsequent lysosome fusion results in degradation of engulfed pathogens making LAP an important process in immune regulation in aging as discussed later.

#### 2.1.2 Endosomal microautophagy

Endosomal microautophagy (eMI) was discovered as a pathway distinct from macroautophagy that delivers cytosolic proteins to late endosomes or multivesicular bodies (MVBs) by a microautophagy-like process ((Sahu et al., 2011); reviewed by Schuck (2020)). eMI is a variant of general microautophagy that does not require the core autophagic machinery but instead depends on the Endosomal Sorting Complex Required for Transport (ESCRT). However, the recruitment of ESCRT is unlike during MVB synthesis (reviewed by Hurley (2008)). eMI is induced upon acute amino acid starvation resulting in rapid degradation independent of the nutrient sensor and classical autophagy regulator, MTOR (Mejlvang et al., 2018). This response was found to be immediate, setting in prior to macroautophagy, with substrates including LC3B, GABARAPL2, and autophagy receptors (Mejlvang et al., 2018). At fly synapses, protein turnover occurs by eMI facilitated by chaperone HSC70-4-dependent membrane deformation while the co-chaperone SGT inhibits microautophagy (Uytterhoeven et al., 2015). HSC70 recognizes synaptic proteins with KFERQ motifs and binds endosomes *via* membrane PS (Sahu et al., 2011). This recognition is distinct from KFERQ recognition during chaperone-mediated autophagy, which involves recognition and import of unfolded proteins into lysosomes (reviewed by Kaushik and Cuervo (2018)).

### 2.2 Secretory autophagy

#### 2.2.1 LDELS

LDELS (LC3-dependent extracellular vesicle loading and secretion) is a form of “secretory autophagy” (SA) that requires the LC3 conjugation machinery for loading cargoes into vesicles which are ultimately released extracellularly. Proximity labeling and extracellular vesicles (EV) proteomics revealed several RNA binding proteins to be the main cargoes of this pathway that also impacts extracellular secretion of non-coding RNAs (ncRNA) (Eng et al., 2021) and small nucleolar RNAs (snoRNA) (Leidal et al., 2020). This highlights a previously unclear role of LC3 in loading cargo into secreted EVs. Yet, how this pathway crosstalks with classical degradative autophagy is still being elucidated. Inhibition of autophagosome maturation, autophagosome-lysosome fusion, or lysosomal acidification each upregulated SA dependent on several ATG proteins and the small GTPase Rab27a. Such EV- and particle-mediated SA facilitates release of autophagic cargo receptors, buffering against their accumulation when classical autophagy is inhibited (Solvik et al., 2022). This highlights an interesting alternative route for maintaining proteostasis by secretory autophagy when autophagosome maturation and lysosome function are impaired.

#### 2.2.2 Unconventional secretion

Secreted proteins usually carry a leader peptide sequence which sorts them to the trans-Golgi network to vesicles destined for the plasma membrane (reviewed by Viotti (2016)). However, proteins with and without leader sequences have been found to bypass the Golgi apparatus to be secreted by pathways together known as unconventional protein secretion (UPS) [reviewed (Ponpuak et al., 2015; Balmer and Faso, 2021)]. Of these UPS pathways, unconventional secretion constitutes sequestration of leaderless proteins into autophagosomes and secretion either by direct binding of the autophagosome with the plasma membrane or by autophagosome fusion with a multivesicular body (MVB) to form an amphisome followed by its fusion with the plasma membrane (Dupont et al., 2011; Zhang et al., 2015). Such secretory autophagosome formation is thought to be facilitated by compartments of UPS (CUPS) in yeast and a yet uncharacterized equivalent in mammalian cells along with Golgi assembly stacking protein (GRASP), ESCRT proteins for MVB formation and sorting, and SNAREs for vesicular fusion (Duran et al., 2010; Manjithaya et al., 2010). Cargo selection, although yet unclear, is thought to involve Vps23, found at CUPS in yeast (Bruns et al., 2011). Cargoes of unconventional secretion include many cytosolic proteins such as IL-1β, IL-18, galectin, tubulin, organellar content, and aggregation-prone proteins (Schweers et al., 2007; Dupont et al., 2011; Ejlerskov et al., 2013; Nilsson et al., 2013; Pallet et al., 2013; Ohman et al., 2014) making UPS a protective pathway to prevent intracellular accumulation, but could also potentially be an important influencer of inflammation.

### 2.3 Recycling autophagy

#### 2.3.1 LANDO

LC3-associated endocytosis (LANDO) begins with recognition of cargo by cell surface receptors like Toll-Like Receptors (TLR) and TREM2 followed by clathrin-mediated endosome internalization. The machinery for the formation of the PI3K complex and LC3 recruitment to the single membrane LANDOsome is similar to that of LAP (Heckmann et al., 2019; Heckmann et al., 2020), but unlike LAP, LANDO has multiple endpoints; LANDOsome fusion with the lysosome followed by ligand degradation and recycling of the cell surface receptors back to the plasma membrane ((Heckmann et al., 2019); reviewed in Pena-Martinez et al. (2022)). The protection offered by LANDO-mediated receptor recycling in microglia in neurodegeneration is discussed in the next section.

## 3 Non-canonical autophagy in aging and age-related diseases

Aging is the number one risk factor for many diseases, and with age, there is a general decrease in efficiency of degradative autophagy, both canonical and NCA (Finkbeiner, 2020; Krause et al., 2022) (Figure 1). Additionally, in what is likely a response to age-associated decreased degradation through the lysosome is the shift to SA (Krause et al., 2022); however, owing to the overlap of the initial steps of autophagosome formation, SA also decreases with age (Gonzalez et al., 2020). Understanding the mechanisms that differentially initiate and regulate NCA will help identify how defects in these pathways contribute to aging and disease.

**FIGURE 2:**
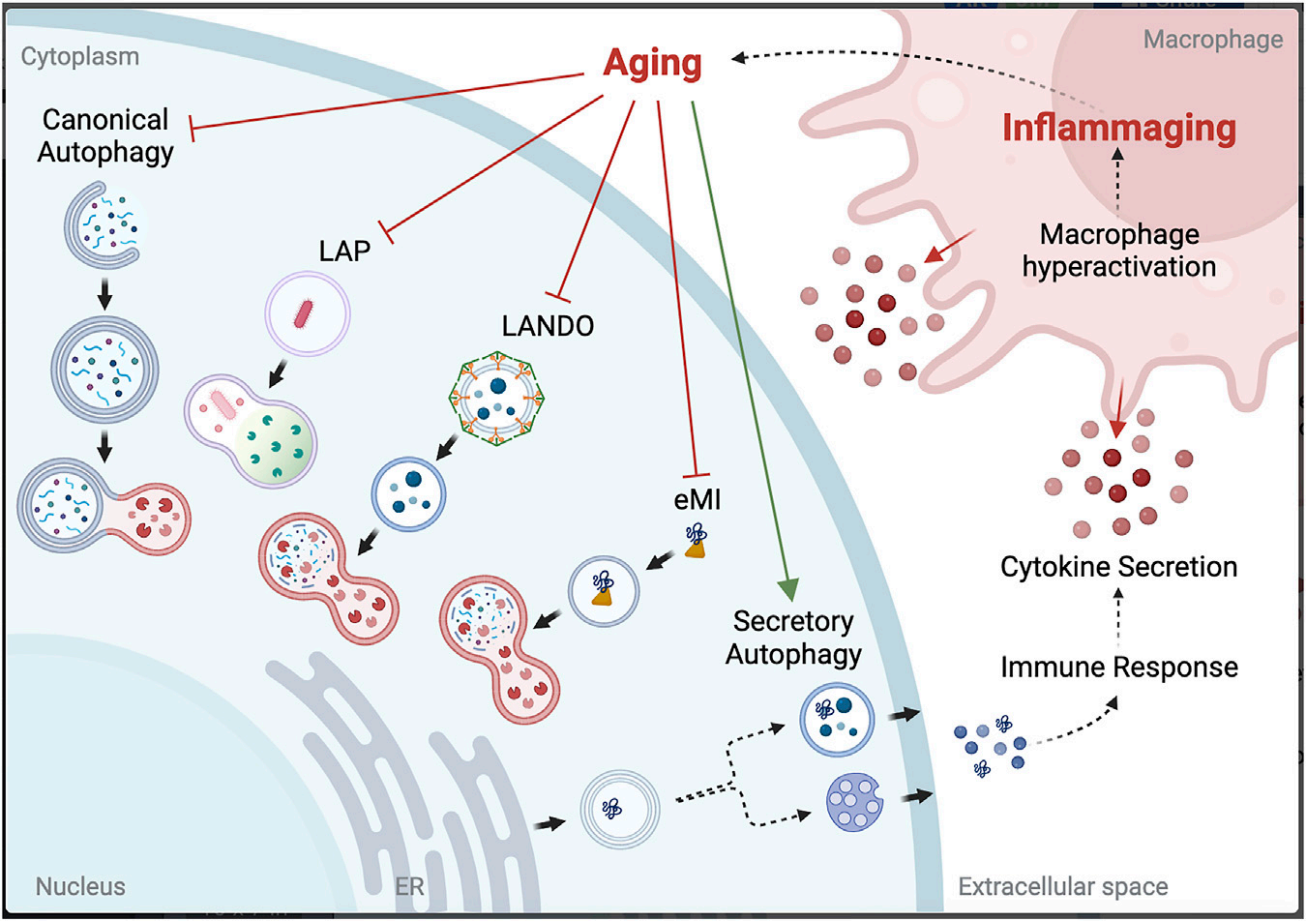
Potential link between autophagy dysfunction and inflammaging. Created with BioRender.com.

One of the defining hallmarks of aging is altered intercellular communication, with a prominent example being “inflammaging”, or the chronic inflammation that further amplifies the aging process (López-Otín et al., 2013). Growing evidence identifies inflammaging as the driver for NCA in aged microglia. SA has been shown to maintain proteostasis when autophagy is inhibited by blocking fusion with the lysosome *in vitro* (Solvik et al., 2022). However, the downstream effect of this is the release of cargo into the extracellular space, and, depending on what was targeted for degradation but is now in the extracellular space, can itself induce an immune response (Tan et al., 2022). Hyperactivation of macrophages will lead to increased phagocytosis of the discarded cargo, bringing it back into the cell to attempt to be cleared by LAP or LANDO. However, if the limitation is at the lysosome, the effort is futile and will lead to deposition of aggregated proteins both intracellularly and in the extracellular space. Thus, chronic inflammation seen with aging is a likely driver for aggregation-associated diseases, including many neurodegenerative diseases (Figure 2).

Cellular senescence is also a hallmark of aging that NCA may have a role in perpetuating. P53-regulated activation of the production and release of exosomes containing miRNA and protein cargoes from senescent cells could worsen senescence-associated secretory phenotypes (SASPs), ultimately resulting in LDELS-driven chronic inflammation (Xu and Tahara, 2013). When investigating NCA pathways in age-associated diseases and potential therapeutic targets, the roles of SA, LDELS, LAP, and LANDO must all be considered.

### 3.1 Alzheimer’s disease

A uniting characteristic of adult-onset neurodegenerative diseases is the abnormal deposition of misfolded aggregated proteins. The age-associated down-regulation of autophagy in the brain suggests autophagy dysfunction is a common mechanism in neurodegenerative disease (Lipinski et al., 2010; Friedman et al., 2012). Alzheimer’s Disease (AD) is caused by neuronal death associated with amyloid beta (Aβ) and tau tangle deposition in the brain. Exploration of canonical autophagy as a therapeutic target for AD has been extensively investigated (reviewed in Guo et al. (2018)), with the targeting of NCA just beginning to surface. Evidence to suggest targeting NCA as a therapeutic is supported by reports that several components that regulate the machinery for LANDO were found to be downregulated in mixed sex and age cohorts of human AD brains compared to matched controls (Heckmann et al., 2020). Experimentally, aged mice (two-years old) lacking the WD domain of ATG16L (specifically required for NCA) showed spontaneous deposits of endogenous Aβ, increased microglial inflammation, and neuronal death in their hippocampi (Heckmann et al., 2020). Further, loss of LANDO leads to a defect in returning the Aβ receptors to the cell surface (Heckmann et al., 2019), so LANDO protects against neuronal loss by improving Aβ clearance in mouse models of AD, owing to the efficient recycling of receptors for Aβ in microglia, including TREM2. This brings to question if inhibition of canonical autophagy to promote LANDO or LDELS would be an appropriate method to consider as an AD therapeutic (Limone et al., 2022).

Further evidence to target NCA for AD therapeutics focuses on LDELS and the role of EV secretion. With age, EV secretion decreases due to the disruption of the endosomal/lysosomal trafficking pathway involved in Aβ metabolism. In a non-human primate study, the contribution to age-associated intraneuronal accumulation of Aβ was partially due to Aβ build-up in EVs. Intraneuronal accumulation of Aβ precedes extracellular Aβ depositions, and the experimental downregulation of autophagosome formation enhanced EV secretion to ameliorate intracellular Aβ accumulation, although there was no success in clearing the extracellular Aβ. Understanding the spatiotemporal transition from intracellular to extracellular depositions may delineate the connection to the age-associated decrease of autophagy-related protein levels that precedes Aβ deposition (Koinuma et al., 2021).

### 3.2 Parkinson’s disease

Parkinson’s Disease (PD) is characterized by neuronal death associated with α-synuclein deposits in the brain. The age-associated loss of autophagy in neurons does not drastically affect the total amount of soluble α-synuclein, suggesting the proteasome is the preferred degradative pathway for α-synuclein, and autophagy would only be activated to clear the aggregated α-synuclein (Ebrahimi-Fakhari et al., 2011). This suggests that autophagy of α-synuclein in neurons does not greatly contribute to the degradation of the protein until it becomes pathological (α-synuclein structure changes or aggregation) and overloads the system (Choi et al., 2022).

The investigation of the specific role of NCA in PD is very undeveloped, although there have been clues to the involvement of SA historically. For instance, it has been demonstrated that α-synuclein is secreted from neurons in PD models (Kim et al., 2013), and that this secretion is a driver of the disease because of the impact it has on neighboring cells. This secretion may be a response to decreased degradative autophagy (Cuervo et al., 2004) by switching to SA to maintain proteostasis in neurons, similar to the mitochondrial SA in cardiomyocytes shown by Huang et al. (2018). Disruption of the canonical autophagic pathway seems to drive SA, indicated by TPPP-p25α’s α-synuclein aggregation properties that also prevents maturation of autophagosomes into autolysosomes by limiting mobility (Ejlerskov et al., 2013). This begs the question if canonical autophagy and SA coexist or if SA is meant as a last resort protective response to the loss of the lysosomal degradation pathway.

### 3.3 Infection and immunity

Responding to infections and inducing an immune response is heavily supported by efficient killing and clearance of pathogens and directing proinflammatory responses by LAP and LANDO in systemic macrophages and dendritic cells in the brain (Heckmann et al., 2017). LC3 recruitment to phagosomes enhances antigen presentation by MHC class II molecules, and the failure of fungal antigen presentation by MHC class II molecules was seen in both mouse and human macrophages when LAP was inhibited (Ma et al., 2012; Romao et al., 2013; Jülg et al., 2020). This could explain why the decreased efficiency of LAP seen with age could make the elderly more susceptible to infectious diseases (Inomata et al., 2020).

Control of the inflammatory response after an infection is as important as modulating an immune response, and NCA has a suggested role in this control. Mitochondria are found to be cleared independently of lysosomal degradation in HeLa cells harboring knockouts of the ATG8 conjugation machinery (ATG7, ATG5, and ATG3). The SA pathway clears mitochondria *via* their extracellular release by a process defined as Autophagic Secretion of Mitochondria along with concurrent increased pro-inflammatory cytokine release from recipient cells (Tan et al., 2022). This study highlights the role of ATG8 lipidation in suppressing inflammatory responses by preventing inflammation-inducing SA of mitochondria.

### 3.4 Cancer

Age is an associated risk factor for many cancers as well, and autophagy in established tumor cells and the supporting cells in the tumor microenvironment is often hyperactivated to support the increased metabolic demand (Sousa et al., 2016; Kimmelman and White, 2017). Therefore, it is not surprising that we are beginning to discover that tumor and associated cells have also adapted the use of the NCA pathways to support tumor growth and progression. For instance, LAP in macrophages that clears dead cancer cells actually helps to suppress an inflammatory response against tumor cells, leading to immune tolerance of these mutant cells (Asare et al., 2020).

However, inhibition of canonical autophagy as a therapeutic must consider the downstream effects of a broad inhibition. As mentioned above, inhibition of autophagy tends to drive NCA. This is particularly detrimental when activating SA, since increased SA is associated with increased cancer proliferation (Gonzalez et al., 2020). While much more work must go into this investigation, in theory, inhibiting canonical autophagy would push the cancer cell to increase exosome release of dangerous ncRNA that perpetuates tumor progression through the uptake of oncogenic exosomes by neighboring cells (Eng et al., 2021). Similar effects could be seen when expelled unhealthy mitochondria are taken up by recipient cells (Tan et al., 2022). With evidence that suppression of LAP has anti-tumor effects (Cunha et al., 2018) and the risk of driving oncogenesis *via* SA, NCA may become a more attractive cancer therapeutic target than canonical autophagy.

## 4 Conclusion and future prospects

The role of NCA in aging and age-related diseases is still under intense investigation. To name a few, preliminary studies have defined roles for LANDO and SA in neurodegenerative diseases, LAP, LANDO, and SA in infection and immune responses, and LAP and SA in cancer, but many questions remain to be answered. It is still not clear how cargo is recruited for NCA, whether NCA and canonical autophagy coexist, if differential signals direct the decision to complete canonical versus NCA, and whether the cell has a preference for either type. Alternatively, NCA may only be initiated when canonical autophagy cannot meet cellular requirements, and thus becomes the dominant response for cargo clearance. Furthermore, the molecular pathways and vesicular trafficking in SA are not fully described, but canonical autophagy machinery is required for the initiation. So, if the same machinery is needed, but there are different outcomes, what determines if degradation occurs in the lysosome or if SA is induced? Moreover, with so many pathways to deliver cargo to the lysosomes for degradation, does everything come down to functional lysosomes? This seems to be the case, since the switch from degradation to SA does not solve the overall problem in neurodegenerative diseases, but instead seems to exacerbate the pathology by inducing a vicious cycle that propagates inflammation (Solvik et al., 2022; Tan et al., 2022). Finally, the most important question is how we can harness NCA to use for prevention, prognosis, or therapeutics for aging and age-associated diseases.
